# Genetic dissection of the natural variation of ovule number per ovary in oilseed rape germplasm (*Brassica napus* L.)

**DOI:** 10.3389/fpls.2022.999790

**Published:** 2022-09-13

**Authors:** Muslim Qadir, Lei Qin, Jiang Ye, Nazir Ahmad, Xinfa Wang, Jiaqin Shi, Hanzhong Wang

**Affiliations:** ^1^Oil Crops Research Institute of the Chinese Academy of Agricultural Sciences, Key Laboratory of Biology and Genetic Improvement of Oil Crops, Ministry of Agriculture and Rural Affairs, Wuhan, China; ^2^Hubei Hongshan Laboratory, Wuhan, China

**Keywords:** ovule number per ovary, genome-wide association study, transcriptomic analysis, QTLs, candidate genes, phytohormones, *Brassica napus*

## Abstract

Oilseed rape is one of the world’s largest oil and industrial crops, providing humans with various products, such as vegetable oil and biofuel. Ovules are the direct precursors of seeds, and ovule number per ovary (ONPO) largely determines seed number per fruit that affects both yield and fitness of seed crops. The ONPO shows wide variation in oilseed rape, whereas the underlying genes and mechanisms are poorly known. The present study performed the genetic, physiological and transcriptomic analyses of ovule number per ovary using an association panel and the extreme lines. The ONPO of 327 accessions planted in four environments showed a large variation from 19.2 to 43.8, indicating a great potential for the further genetic improvement of ovule number. The genome-wide association study (GWAS) identified a total of 43 significant SNP markers. Further, these SNPs were integrated into 18 association loci, which were distributed on chromosomes A01, A03, A06, A07, A09, C01, C03, C06, C07, and C09, explaining 4.3–11.5% of the phenotypic variance. The ONPO decreased as their appearance order on the inflorescence and was associated with the level of several types of endogenous phytohormones but not related to leaf area and photosynthetic rate. Comparative transcriptomic analysis identified a total of 4,449 DEGs enriched in 30 classes, including DNA, RNA, protein, signaling, transport, development, cell wall, lipid metabolism, and secondary metabolism. Nearly half of DEGs were involved in the known pathways in regulating ovule number, of which 12 were homologous to know ovule number regulating genes, indicating a strong link between the identified DEGs and ovule number. A total of 73 DEGs were located within the genomic regions of association loci, of which six were identified as candidates based on functional annotation. These results provide useful information for the further genetic improvement of ovule and seed number in oilseed rape.

## Introduction

*Brassica napus* L. (AACC, 2*n* = 38) is an allopolyploid (AACC) species generated from an interspecies crossing between *B. rapa* (AA, 2*n* = 20) and *B. oleracea* (CC, 2*n* = 18) around 7,500 years ago (Chalhoub et al., 2014; Wang et al., 2020; Bilgrami et al., 2022). Oilseed rape is one of the most important oilseeds crops worldwide after soybean that provides high-quality nutrients and nutraceuticals to humans and animals and biofuel for industrial production (Yang et al., 2017; Khan et al., 2019). With the increasing demand for edible oil and biofuel due to population growth, it is an urgent need to improve seed yield per unit area in *B. napus* (Hu Q. et al., 2017; Ahmad et al., 2021; Li et al., 2021).

Under the same planting area, seed yield per unit area is determined by seed yield per plant. In *B. napus*, seed yield per plant is a complex quantitative trait that is determined by three components: silique number per plant, seed number per silique, and seed weight in oilseed rape (Clarke and Simpson, 1978; Li et al., 2015). Of these, the seed number per silique relies on the ovule number per ovary (ONPO), the proportion of ovules to be fertilized and the proportion of fertilized ovules to develop into seeds (Yang et al., 2017; Yuan and Kessler, 2019). Ovules are the direct precursors of seeds containing the female gametophytes, which are fecundated during pollination to inaugurate seed development (Yuan and Kessler, 2019). Therefore, increasing the number of ovules per flower has become an important strategy for improving seed crop yield and addressing food security (Jiao et al., 2021). The number of ovules per flower varies considerably across different species and even among the different accessions of the same species (Burd et al., 2009; Khan et al., 2019). Ovule number per ovary is determined by the ovule initiation process and is significantly affected by flower size and position (Gomez et al., 2018; Yuan and Kessler, 2019), nutrient availability (Strelin and Aizen, 2018), and phytohormones levels (Barro-Trastoy et al., 2020; Qadir et al., 2021). Although much research into seed number per silique has been reported in *B. napus* (Yang et al., 2017; Khan et al., 2019; Zhu et al., 2020), few were performed on ovule number per ovary (Khan et al., 2019; Jiao et al., 2021). Therefore, little is known about the natural variation of ovule number per ovary in oilseed rape germplasm.

Although none of the ovule number QTLs in *Brassica* has been cloned, nearly one hundred ovule number regulating genes have been reported in plants (mainly from *Arabidopsis*), which can be used as the reference for *Brassica*, due to their close relationship (Qadir et al., 2021). The systematic summarization of these ovule number genes showed that it is governed by an integrated genetic and phytohormones network where AUX, BR, and CK are the positive regulator of ovule number, whereas GA acts negatively on it (Nemhauser et al., 2000; Bencivenga et al., 2012; Yuan and Kessler, 2019). For example, *PIN1* is one of the eight transmembrane auxin transporters in *Arabidopsis* which is involved in polar auxin transport and required for ovule primordia formation (Benková et al., 2003; Galbiati et al., 2013). The *pin1* mutant shows reduced auxin transport activity and multiple growth and development defects, including reduced ovule number (Okada et al., 1991).

Genome-wide association analysis can detect the causal loci underlying complex quantitative traits at the whole-genome level, which may contain several to hundreds of genes dependent on the LD decay at these loci. Transcriptomic analysis can quantify gene expression level and identify the differentially expressed gene in the given tissues at the specific stage, which may be upstream causal genes or downstream target genes in regulating trait variation. Numerous studies have demonstrated that the integration of genome-wide association and transcriptomic analysis has become an efficient strategy for identifying candidate genes underlying complex quantitative agronomic traits (Wang et al., 2020; Helal et al., 2021). For example, by GWAS of silique length and transcriptomic analysis of silique wall at 15-DAF, *BnaA9.CYP78A9* was successfully identified as the causal gene for a major association loci *qSL.A09-3* (Hussain et al., 2021). The present study aims to dissect the genetic, physiological, and molecular basis for the natural variation of ovule number per ovary in oilseed rape (*Brassica napus* L.). These results will provide a solid basis for further gene cloning and genetic improvement of ovule number in oilseed rape.

## Materials and methods

### Plant materials and field trials

The association population was composed of 327 oilseed rape accessions (Li et al., 2020). The field experiments were conducted in four environments, including May 2020 at Ping’an district (36.47°N, 102.09°E) in Haidong city of Qinghai province, Oct 2020 at Yangluo (31.84°N, 114.8°E), and Oct 2021 at both Wuchang (30.35°N, 114.33°E) and Yangluo district in Wuhan city of Hubei province. The field planting followed a randomized complete block design with three biological replications. The field management was conducted according to the local standard practices.

### Investigation of ovule number

At the beginning of flowering, three buds were collected from the bottom of the main inflorescence of five plants randomly, resulting in 15 buds for each replication. In total, 58,860 buds (4 environments × 327 lines × 3 replications × 15 buds) were sampled. The sampled buds were fixed in FAA, of which 10 randomly selected buds were dissected for further observation. The calyx and petals of sampled bud were removed by a dissecting needle and taken out of the ovary. The ovaries were kept in 2 ml Eppendorf tubes containing 90% alcohol solution twice and then washed with ddH_2_O. The trichloroacetaldehyde hydrate solutions were added in a small amount to submerge the sample and make the ovaries transparent. The ovaries were kept in a transparent solution for 12 h to 3 days’ maximum and then transferred to glass slides under the microscope (SZX2-ILLT, Olympus Corporation, Japan). The ovule numbers were counted manually following previously described methods (Yang et al., 2017; Ali, 2018; Yu et al., 2020).

### Observation of ovule number per ovary in different positions

To investigate whether ovule number varied with the physical position of buds on the inflorescence. The ovule number variation from the bottom to the top of the inflorescence was investigated using two representative extreme lines (Yang et al., 2017). Briefly, the ovule number data was investigated from five consecutive buds (such as 1–5, 6–10) before flowering. The buds from the bottom to the top of the main inflorescence of the representative plants were sampled.

### Detection of phytohormones

To investigate whether the ovule number variation is associated with the concentrations of endogenous phytohormones, the ovaries at the ovule initiation stage were measured for extreme lines. The buds of 0.5–1 mm length were dissected by hand under a stereomicroscope within the required temperature (Vera-Sirera et al., 2016; Gomez et al., 2018), and the obtained ovaries were mixed equally to generate two pools with three biological replications. The quantification of specified hormones including; Abscisic Acid (ABA), Benzyl Adenine (BA), Gibberellic Acid (GA4), Indole-3-Acetic Acid (IAA), Indole-3-Butyric Acid (IBA), Jasmonic Acid (JA) and Salicylic Acid (SA) were carried out on the phytohormones platform at Huazhong Agricultural University, Wuhan, China.

### Measurement of leaf area and photosynthetic rate

At the early flowering time, ten mature leaves were collected from five representative plants of the extreme lines and transferred to the laboratory. The procedure for leaf area measurement was described as in the previous studies (Hu M. et al., 2017; Li et al., 2020). A portable photosynthesis system (LI-6800XT, LI-COR) was used to measure the photosynthetic parameter of the extreme lines in the field between 9:30–11:00 and 14:30–16:00 (Li et al., 2019; Wang et al., 2020).

### Genome-wide association analysis

The *Brassica* 50 K SNP array^1^ was used for the genotypic analysis of the association population, which contains 45,707 SNP markers. The parameters were set as a missing rate ≤0.2, heterozygous rate ≤0.2, and minor allele frequency >0.05 to examine SNP data using Illumina Bead Studio genotyping software.^2^ The probe sequences of these SNP markers were compared to the *B. napus* Darmor-bzh reference genome to identify their physical positions (Chalhoub et al., 2014). The four statistical models were used for the association study, including the general linear model (GLM) with controlling for population structure (Q) and principal component analysis (PCA), the mixed linear model (MLM) controlling for both Q and PCA with relative kinship (K) according to Liu et al. (2016). The GWAS analyses were performed using TASSEL v.5.2.77 software. The threshold for the significantly associated SNP markers was set to *P* < 4.08 × 10^–5^ [*P* = 1/21242, −log_10_ (*P*) = 4.33] as previously described (Liu et al., 2016; Li et al., 2020).

### Ribonucleic acid sequencing and transcriptomic analysis

The ovaries at the ovule initiation stage were dissected from 0.5 to 1 mm buds by hand under a stereomicroscope at the required temperature. The obtained ovaries were mixed equally to generate two pools with three biological replications (M1-M3 and L1-L3). According to the manufacturer’s procedure, a Plant RNA Mini Kit was used to isolate total RNA from each sample (Tiangen, Inc., China). The Oebiotech company performed cDNA library construction and Illumìna sequencing using an Illumìna HìSeq™ 2,500 platform.

The low-quality, low-complexity, and repetitive raw reads were sorted out, and only the clean reads that passed quality control were subjected to further analysis. The sequences were mapped to the reference genome of Darmor as previously described (Hussain et al., 2021). The false discovery rate (FD^–^ RD ≤0.05) and the *p*-value ≤0.005 were set as a threshold to identify the deferentially expressed genes (DEGs). Moreover, these paired-end sequencing reads were uploaded to NCBI with accession number PRJNA820145.

### Validation of deferentially expressed genes through qRT-PCR

Ten DEGs randomly chosen were subjected to qRT-PCR validation using the same RNA samples used for sequencing. The M-MLV reverse transcriptase (Promega) was used to synthesize cDNA containing total RNA (4 μg) and oligo (dT) primers, as described in the company instructions. Bio-Rad CFX96 real-time detection systems were used for qRT-PCR analyses with three replicates. The 2^–ΔΔ*Ct*^ method was adopted to evaluate the relative expression of target genes, with the *B. napus* ACTIN2 as an internal control (Li et al., 2021). The details of these gene-specific primers used for qRT-PCR are listed in Supplementary Table 1.

### Identification of candidate genes

Based on the physical distance of LD decay, the genes within 500 kb up and downstream of significantly associated SNP loci were considered (Li et al., 2020). Some of these genes were homologous to the known ovule number regulating genes in plants (Qadir et al., 2021), which were identified as candidates. In addition, some of these genes showed significantly differential expression between the more and less-ovule lines, which were also identified as candidates.

### Statistical analysis

The correlation coefficient was calculated using the CORR procedure implemented in the SAS software 8.0 (SAS Institute, Inc., 2000, Cary, NC, United States). Broad-sense heritability was calculated as *h*^2^ = σ_g_^2^/(σ_g_^2^ + σ_ge_^2/^n + σ_e_^2^/nr), where σ_g_^2^, σ_ge_^2^, and σ_e_^2^ are the variances of genotype, genotype by environment and error, respectively, while n and r are the number of environments and replicates, respectively. The Excel statistical functions CHISQ.TEST and T.TEST were used to obtain the significance level (P_x_^2^_–test_ and P*_t_*_–test_) of the degree-of-fit and differences (Meng et al., 2015). The frequency distributions of ONPO investigated in four environments were constructed using Minitab 9.1 software.

## Results

### Phenotypic variation of ovule number per ovary

**FIGURE 1:**
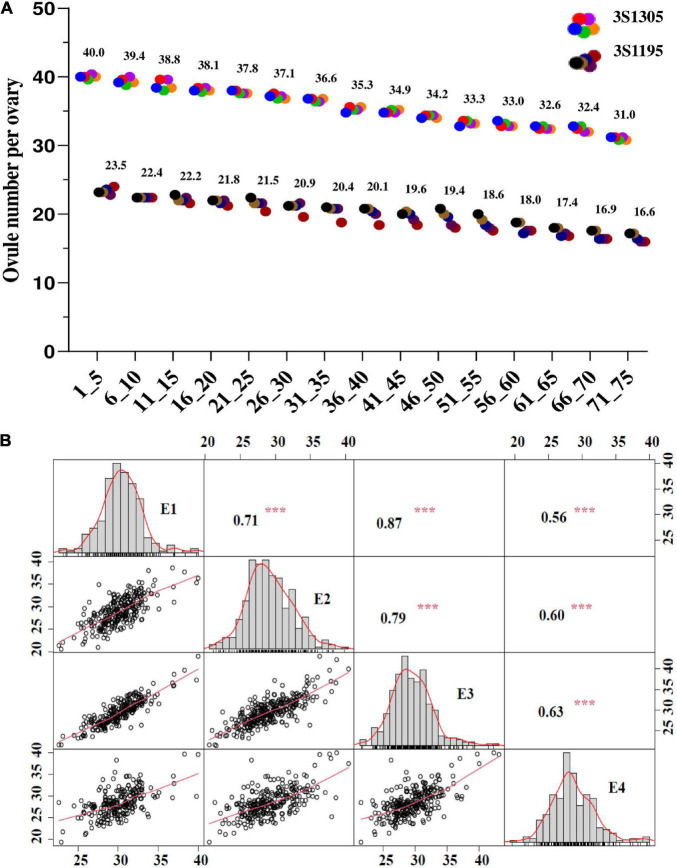
The phenotypic variation and correlation of ONPO in an association panel of oilseed rape across four investigated environments. **(A)** The ONPO in the different positions of the main inflorescence from two representative lines (3S1305 and 3S1195). The horizontal and vertical axes show the bud order and ONPO, respectively. **(B)** The frequency distribution and correlation of ONPO in four investigated environments. The diagonal line plot shows the ovule number frequency distribution. The numerals above the diagonal line are Pearson-correlation coefficient values between environments, and diagrams below the diagonal line indicate the scatter plots of the ovule number. *** Represents the significance level of *P* = 0.001.

The ONPO of two representative lines (3S1305 and 3S1195) were investigated from every five buds sampled from bottom to top of the main inflorescence. The results showed that the calculated ONPO of the two lines ranged from 40.0 to 31.0 and 23.5 to 16.6, respectively (Figure 1A), showing an obvious continuous downward trend in order of occurrence/differentiation. Therefore, the ONPO of the association population was investigated from the buds on the bottom of the main inflorescence.

The descriptive statistics of ONPO of the association population in four environments were presented in Table 1. In general, the ONPO of 327 lines in the association panel varied from 19.2 to 43.8 across the four investigated environments, displaying more than two-fold variation. The coefficient of variation of ONPO in the four environments was 8.91, 12.64, 11.64, and 11.50%, respectively (Table 1). In general, the high correlations of ONPO were observed between pair-wise environments (*r*^2^ = 0.71, 0.79 and 0.87) except for 2020QH (*r*^2^ = 0.56, 0.60 and 0.63). The phenotypic frequency distribution analysis showed that the ONPO was almost normally distributed in each environment, indicating a quantitative inheritance suitable for QTL mapping (Figure 1B). Furthermore, the analysis of variance showed that the variation of ONPO in this association panel was primarily attributed to the genotypes (Supplementary Table 2). The calculated broad-sense heritability from variance components was 91.9%, indicating the high stability of this trait suitable for genetic study.

**TABLE 1:** Descriptive statistic of ovule number per ovary trait in four investigated environments.

Environments	Mean ± SE	SD	Min	Max	Variance	CV (%)	Kurtosis	Skewness
20QH	30.14 ± 0.13	2.69	22.20	40.20	7.22	8.91	1.25	0.26
20YL	29.11 ± 0.12	3.54	19.70	42.00	12.52	12.16	0.22	0.34
21WC	29.64 ± 0.11	3.45	20.30	43.80	11.89	11.64	1.25	0.64
21YL	28.50 ± 0.14	3.28	19.22	40.80	10.75	11.50	1.32	0.67

20QH, 20/21YL and 21WC are the abbreviations of the 20 Qinghai, 20/21 Yangluo and 21 Wuchang environments, respectively.

### Genome-wide association study of ovule number per ovary

**FIGURE 2:**
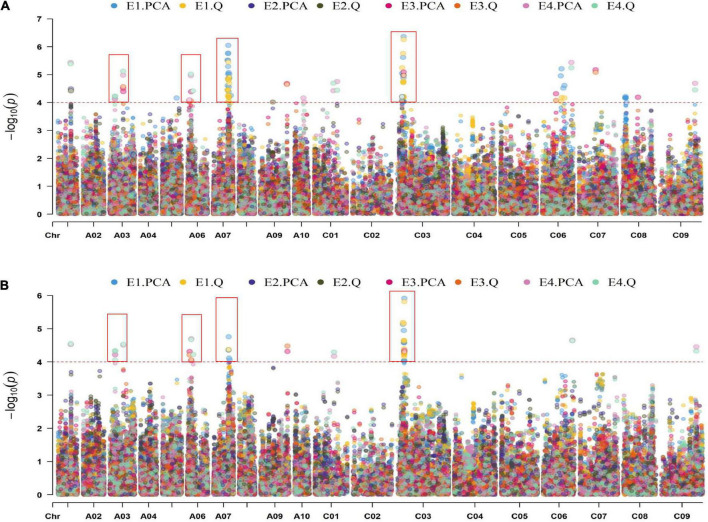
Manhattan plot of GLM **(A)** and MLM **(B)** in four environments. The horizontal and vertical axes show the length of each chromosome and –log_10_*P*-value, respectively. The scatters on the figure show the –log_10_(*P*-values) of the corresponding SNP markers. The dotted lines on the figure showed the threshold for the –log_10_(*P*-value). The different colors distinguished the different environments and models, as shown in the legends.

In total, 21,242 SNPs data previously generated in our group were used for the association study (Figure 2 and Supplementary Figure 1). A total of 43 SNP loci were significantly associated with the ONPO (Supplementary Table 3). Specifically, 28, 1, 6, and 7 loci were detected in 20QH, 21WC, 20YL, and 21YL, respectively. It should be noted that many SNP loci were detected by multiple models and environments, suggesting their reliability. After the integration of close SNP loci within 500 kb, a total of 18 QTLs were obtained, which were distributed on chromosomes A01, A03, A06, A07, A09, C01, C03, C06, C07, and C09, explaining 4.3 to 11.5% of the phenotypic variance (Table 2).

**TABLE 2:** The details of 18 association loci of ovule number per ovary.

QTLs	Model	Marker	Position	−log10 (*P*)	Ad_ effect	PVE (%)	ENV
*qONPO.A01-1*	1,2,3,4	Bn-A01-p17498057	14,947,415	−0.734609145	0.57822	9.3	E4
*qONPO.A01-2*	1,2	Bn-A01-p17118647	15,506,771	−0.652511091	0.88558	6.5	E3
*qONPO.A03-1*	4	Bn-A03-p7234862	6,510,685	−0.636440903	0.85913	7.8	E4
*qONPO.A03-2*	1,2,3,4	seq-new-rs32414	16,137,692	−0.709442121	−1.0347	8.7	E4
	2	seq-new-rs32414	16,137,692	−0.659806462		6.9	E2
*qONPO.A06-1*	1,2,3,4	seq-new-rs23621	4,708,433	−0.671900437	0.98819	8.6	E4
	1	Bn-A06-p5616164	5,062,564	0.000040773		4.3	E2
*qONPO.A06-2*	2	seq-new-rs39246	7,101,109	−0.645020967	/	6.1	E4
*qONPO.A07-1*	1,2	Bn-A07-p15591535	17,487,704	−0.74167809	0.54459	9.3	E1
*qONPO.A07-2*	1,2,3,4	seq-new-rs40795	18,145,354	−0.781835256	−0.39028	10.3	E1
*qONPO.A07-3*	1,2	Bn-A07-p17225853	19,141,651	−0.700557723	−0.16366	8.6	E1
*qONPO.A09-1*	1,2,4	seq-new-rs41467	31,832,091	−0.651051124	1.15762	7.0	E2
*qONPO.C01-1*	1,2	seq-new-rs36223	22,560,520	−0.671818418	−0.34682	7.8	E4
*qONPO.C01-2*	1,2	seq-new-rs42691	27,093,843	−0.677173624	0.21882	8.1	E4
*qONPO.C03-1*	1,2,3,4	seq-new-rs30160	6,454,037	−0.710287855	−0.38299	9.9	E1
*qONPO.C03-2*	1,2,3,4	Bn-scaff_18322_1-p806079	8,231,877	−0.771941275	−0.060327	11.5	E1
	1,2,3	Bn-scaff_18322_1-p806079	8,231,877	−0.706911562		7.5	E2
*qONPO.C06-1*	1	seq-new-rs43825	22,653,417	−0.71681982	/	7.6	E1
*qONPO.C06-2*	1,2,3,4	seq-new-rs45976	34,229,155	−0.73531806	2.70296	9.3	E4
*qONPO.C07-1*	1,2	seq-new-rs28170	19,782,297	−0.713787513	/	7.5	E2
*qONPO.C09-1*	1,2,3,4	Bn-scaff_19899_1-p12334	41,095,875	−0.648738222	/	6.7	E4

E1–E4 are the codes of the above four environments 20QH, 20YL, 21WC and 21YL, respectively. 1–4 are the four models GLM-PCA, GLM-Q, MLM-PCA, and MLM-Q, respectively.

**FIGURE 3:**
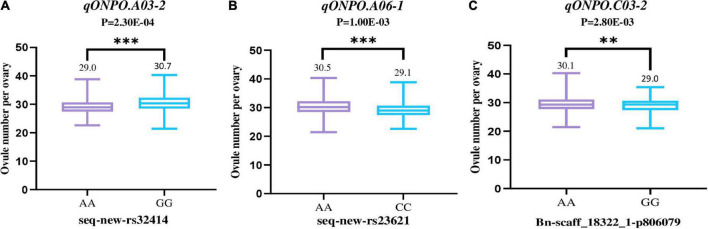
The phenotypic effects of three repeatable association loci **(A–C)**. The horizontal axis shows the haplotype of the peak SNP for the association loci. The vertical axis shows the ONPO. The violin diagram in the figure shows the mean and range of ONPO for each haplotype. The *P*-values of the *T*-test between the ONPO of corresponding haplotypes were also shown at the top of figure. ** and *** represent the significance level of *P* = 0.01 and 0.001, respectively.

It should be noted that two associated loci (*qONPO.A03-2* and *qONPO.A06-1*) were repeatedly detected in 20YL and 21YL environments, and one (*qONPO.C03-2*) was repeatedly identified in 20QH and 21YL environments. Therefore, they may represent important targets for marker-assisted selection and further gene cloning. For *qONPO.A03-2*, the two variant bases A and G of peak SNP seq-new-rs32414, respectively accounted for 75.5 and 24.5%. The G allele’s mean ONPO (30.7) was significantly greater than that (29.0) of the A allele with *P* = 2.30E^−4^ (Figure 3). For *qONPO.A06-1*, the two variant bases A and C of peak SNP seq-new-rs23621, respectively, accounted for 25.2 and 74.8%. The A allele’s mean ONPO (30.5) was significantly greater than that (29.1) of the C allele with *P* = 1.00E^−3^. For *qONPO.C03-2*, the two variant bases A and G of peak SNP Bn-scaff_18322_1-p806079, respectively, accounted for 82.5 and 17.5%. The A allele’s mean ONPO (30.1) was significantly greater than that (29.0) of the G allele with *P* = 2.80E^−3^.

Furthermore, relative to the leading SNPs of these association loci, a total of 1971 annotated genes were located within 500 kb or LD statistic *r^2^* > 0.2. A dozen QTL for ONPO have been reported through linkage or association analysis in oilseed rape (Okada et al., 1991; Jiao et al., 2021). To determine their positional relationship with the association loci identified in the current research, comparative QTL analysis was conducted based on the reference genome of Darmor_V4.1.^3^ The results indicated that all 18 association loci are not overlapped with the published QTLs of ONPO, suggesting that they are all novel loci.

### Screening of lines with extreme ovule number

**FIGURE 4:**
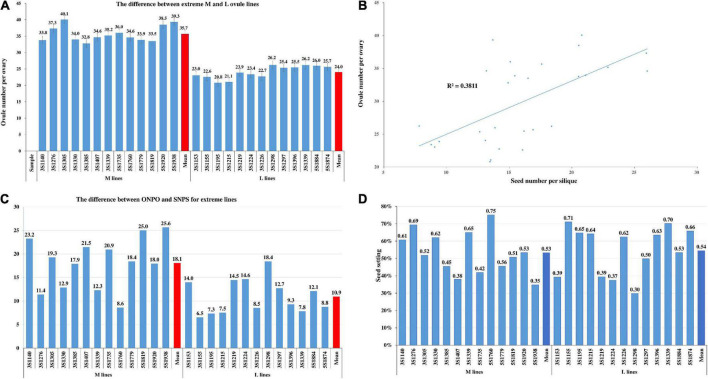
The ONPO of extreme lines and its relationship with SNPS. **(A)** The of ONPO between 13 more- and 13 less-ovule lines. The horizontal and vertical axes show the line number and ovule number per ovary, respectively. The columns of different heights indicate the number of ovules. The bar on each column represents the standard deviation. **(B)** The correlation between ONPO and SNPS of 26 extreme lines. The horizontal and vertical axes show SNPS and ONPO, respectively. The positions of 26 dots show the corresponding ovule and seed number of them. The 26 pairs of data were used to fit the trend line with *R*^2^. **(C)** The difference between ONPO and SNPS of extreme lines. **(D)** The seed-setting rate of 26 extreme lines.

In order to further comparative study, a total of 26 representative lines with more or less ovule were selected based on their ONPO data in four environments. The ONPO of 13 more- and 13 less-ovule lines ranged from 32.8 to 40.1 and from 20.8 to 26.2, respectively, and the mean of the former (35.7) was significantly larger than that of the latter (24.0) with *P* = 8.20E^–13^ (Figure 4A). As expected, the ONPO of these extreme lines displayed a significant (*P* = 2.78E^–13^) positive correlation with SNPS, with *r* = 0.617 (Figure 4B). The difference between ONPO and SNPS of the M and L line ranged from 8.6 to 25.6 and 6.5 to 18.4, respectively (Figure 4C), and the mean of the former (18.1) was significantly larger than that of the latter (10.9). The calculated seed-setting rate of the M and L lines varied from 34.8 to 75.1% and from 29.8 to 71.1%, respectively. The average seed-setting rate of M and L lines (53.3 and 54.5%) had no significant difference, indicating that the genetic control of ONPO and seed-setting rate should be different (Figure 4D).

### Comparative physiological study between extreme lines

To obtain insight into the physiological processes that affect ONPO, the leaf area, leaf photosynthetic rate, and phytohormones content were investigated and compared between the extreme lines.

**FIGURE 5:**
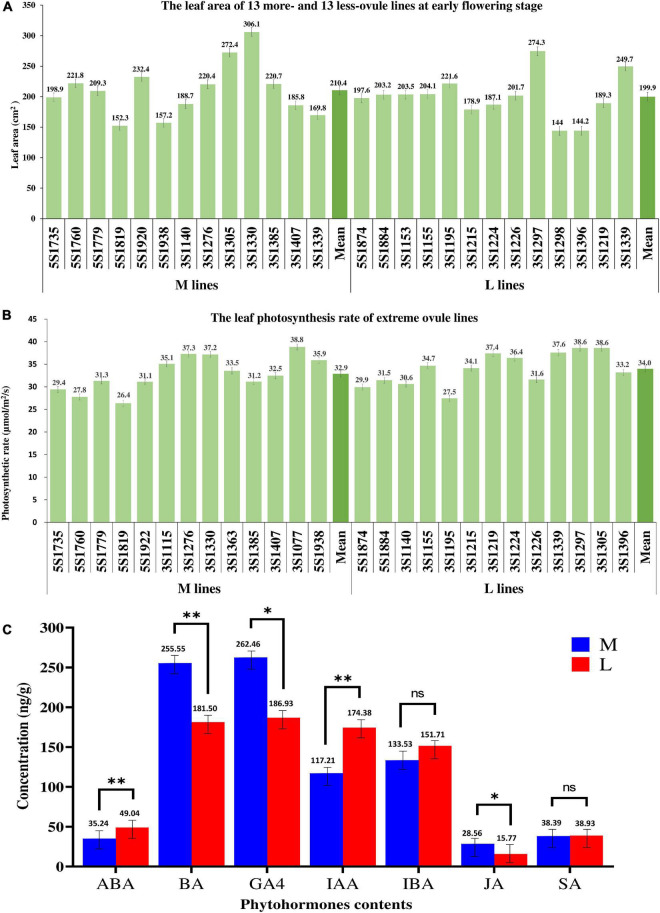
The comparison of three physiological indexes between more- and less-ovule lines. **(A,B)** The leaf area leaf photosynthetic rate of 13 more- and 13 less-ovule lines. The horizontal axis shows the line number, and the vertical axis shows **(A)** leaf area or **(B)** leaf photosynthetic rate. The columns of different heights show the leaf area and photosynthetic leaf rate of different lines. The numerals and bars on each column represent the mean and standard deviation, respectively. **(C)** Comparison of phytohormones content between more- and less-ovule lines. The horizontal and vertical axes show the phytohormone types and their content, respectively. The columns of different heights show the contents of different lines. The numerals and bars on each column represent the mean and standard deviation, respectively. * and ^**^ represent the significance level of *P* = 0.05 and 0.01, respectively.

The photosynthetic rates of 13 more- and 13 less-ovule lines varied from 26.36 to 38.82 mol/m^2^/s and from 27.47 to 38.59 mol/m^2^/s, respectively (Figure 5A). The mean of the former (32.88 mol/m^2^/s) had no significant difference from that of the latter (33.97 mol/m^2^/s). As expected, the correlation between leaf photosynthetic rate and ONPO of these lines was very low and not significant (*r*^2^ = 0.0002).

The leaf area of the 13 more-ovule and 13 less-ovule lines ranged from 152.32 to 306.12 cm^2^ and from 144.03 to 247.34 cm^2^, respectively (Figure 5B). It should be noted that the average leaf area of 13 more-ovule lines (210.40 cm^2^) was not significantly larger than that (199.97 cm^2^) of the 13 less-ovule lines (*P* = 5.13E^–01^). The correlation between leaf area and ONPO of these lines were also low and not significant (*r*^2^ = 0.0054), suggesting that leaf area may not affect ONPO (Supplementary Figure 2).

The contents of BA, GA4, and JA in M lines (255.5, 262.5, and 28.6 ng/g) were significantly higher than those in L lines (181.5, 186.9, and 15.8 ng/g). In contrast, L lines exhibited significantly higher contents of ABA, IAA and SA (49.0, 174.9, and 151.7 ng/g) than M lines (35.2, 117.2, and 133.5 ng/g). There was no significant difference in the contents of IBA and SA between the two types of extreme lines (Figure 5C). These data suggested that the content difference of the above phytohormones should be associated with ONPO difference between M and L lines (Supplementary Table 4).

### Comparative transcriptomic analysis between extreme lines

To investigate how the ovule number difference formed at the molecular level, a comparative transcriptome analysis was performed using the ovaries of M and L extreme lines at the ovule initiation stage. The summary of the transcriptomic data produced by the Illumina sequencing platform is presented in Supplementary Table 5. After filtering out low-quality reads, 143,282,598 and 148,303,824 total reads and 143.28 M to 148.3 M clean reads were obtained from RNA sequencing of three repeats of more- and less-ovule lines, respectively (Supplementary Table 5). The mapped reads rate of the L and M groups varied from 85.5 to 90.9% and from 89.5 to 92.8%, with a mean of 89.0 and 92.0%, respectively. The Q30 percentage of the L and M group ranged from 89.1 to 90.4% and from 89.6 to 90.1%, with a mean of defined 89.9 and 89.8%, respectively. The L and M group’s guanine and cytosine (GC) contents varied from 45.7 to 45.8% and from 45.4 to 46.3%, with a mean of 45.8 and 45.9%, respectively.

**FIGURE 6:**
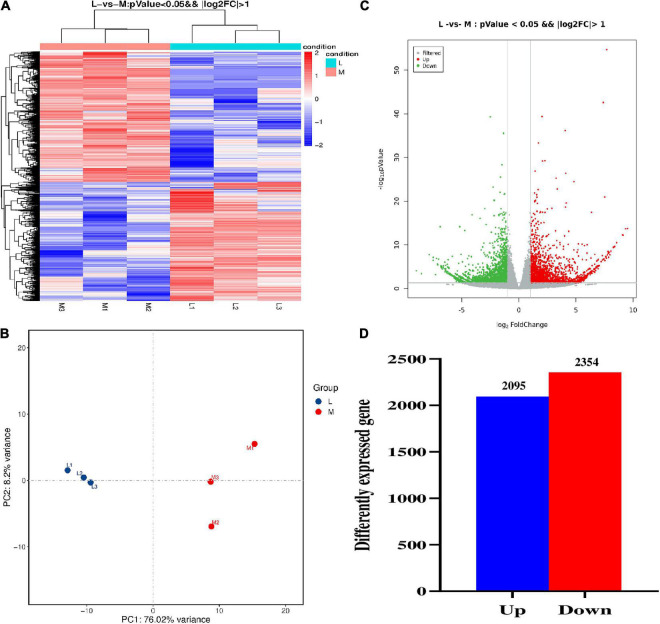
The comparative transcriptomic analysis between more- and less-ovule lines. **(A)** The heat map of DEGs between more- and less-ovule lines. The color column indicates the normalized signal value. The more- and less-ovule lines were distinguished using the different colors. L1 to L3 and M1 to M3 represent the three repeats and less and more-ovule lines. **(B)** Principal component analysis of gene expression level of the three repeats of more- and less-ovule lines. The horizontal and vertical axes show the variance of PC1 and PC2, respectively. The more- and less-ovule lines were distinguished using the different colors. **(C)** The volcano map of expressed genes in more- and less-ovule lines. The horizontal and vertical axes show log_2_ Fold Change and –log_10_(*P*-value), respectively. **(D)** The statistic of DEGs number between more- and less-ovule lines. The horizontal and vertical axes show the up/down pattern and number of DEGs, respectively.

The gene expression density of six samples was similar (Supplementary Figure 3A). As expected, the correlation coefficients among the three repeats of the same group (M or L group) are larger than those between the two groups (M and L) (Supplementary Figure 3B). The FPKM value of each expressed gene was also calculated, and its distribution was similar among the six samples (Supplementary Figure 3C). We carried out the hierarchical clustering analysis (HCA) and principal component analysis (PCA) to differentiate between the two groups. The results showed that the transcriptome of M and L lines was very different (Figures 6A,B). We constructed the volcano plot to determine the significantly expressed genes for the identification of DEGs (Figure 6C). A total of 4,449 DEGs were identified from the 95,791 expressed genes in two groups, containing 2,095 up-regulated and 2,354 down-regulated genes between two types of lines (Figure 6D). To validate the accuracy/reliability of RNA-seq analysis, ten representative DEGs were randomly selected to validate through qRT-PCR analyses. Except for *BnaC07g41610D*, most of the selected genes displayed similar expression patterns with the transcriptomic data, although the fold of change varied between the two methods (Supplementary Figure 4).

**FIGURE 7:**
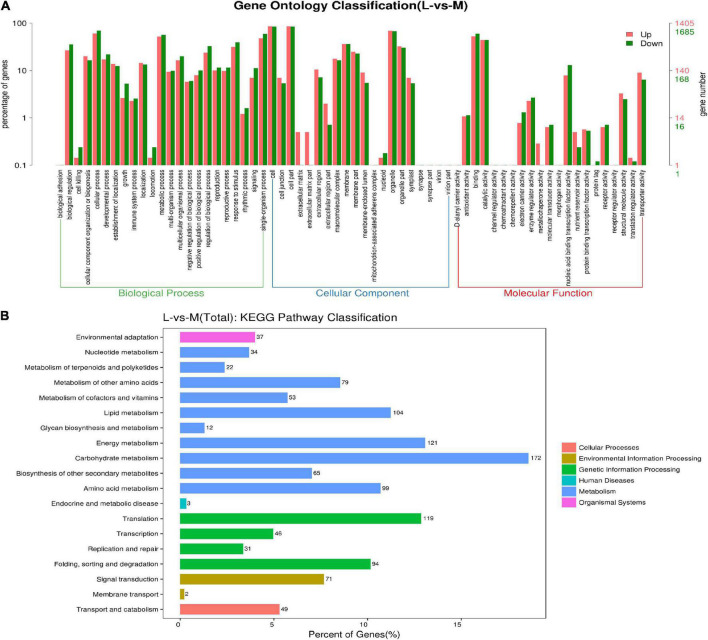
The functional categorization of differentially expressed genes. **(A)** GO enrichment histogram of DEGs. The horizontal axis shows three main categories (biological process, cellular component, and molecular function), each containing several subclasses. The vertical axis shows the number and proportion of DEGs in up and down-regulation. **(B)** KEGG enrichment histogram of DEGs. The horizontal and vertical axes show the number/percent and type of DEGs, respectively.

The functional categorization results of the DEGs were further confirmed *via* gene ontology (GO) and KEGG analysis (Figure 7). A total of 4,449 DEGs were subjected to an enrichment analysis for GO annotation terms. The biological process included adhesion, regulation, biogenesis, cellular and developmental process, reproductive process and rhythmic process, etc. These DEGs were enriched in cell junction, extracellular matrix, nucleotide, organelle, and virion for cellular components. For molecular function, these DEGs were enriched in Antioxidant activity, catalytic activity, molecular structure-activity, transporter, and receptor activity, etc. Moreover, we performed a KEGG enrichment analysis after identifying DEGs in two pools to characterize the DEGs. The results showed that these DEGs were enriched in 19 biological pathways, including environmental adaptation, nucleotide metabolism, carbohydrate metabolism, translation, genetics, biosynthesis of other secondary metabolism, and proteins, etc.

### Characterization of differentially expressed genes

To reveal the molecular mechanisms that involve the DEGs, they were submitted to the super viewer^4^, which displayed the total number and frequency as well as the *P*-value for each class (Supplementary Table 6). The results indicated that the DEGs are involved in diverse metabolic processes, including DNA (57), RNA (540), photosynthesis (PS, 79), transport (176), protein (489), signaling (196), stress (121), cell (114), cell wall (97), hormone metabolism (74), etc.

Of the 540 DEGs in the largest group of RNA classes, 341 (63.1%) were involved in transcription factors, indicating the importance of TF in regulating ovule number. These transcription factors DEGs were from 47 types, including unclassified TFs (35), MYB (33), bHLH (33), Putative transcription regulator (21), Homeobox (21), C2H2 zinc finger (20), AP2/EREBP (15), WRKY (11), bZIP (11), B3 TFs (10), C2C2 (Zn) DOF zinc finger (9), C2C2 (Zn) GATA (9), G2-like TFs (9), MADS box (8), ARR (8), Trihelix (6), Aux/IAA (6), Chromatin Remodeling Factors (5), General Transcription (5), SET-domain (4), C2C2 (Zn) CO-like (4), AS2 (4), Zf-HD (3), YABBY (3), HSF (3), CCAAT box binding factor (3), E2F/DP TFs (3), DNA methyl transferases (3), FHA TFs (2), Global TFs (2), Histone acetyltransferases (2), sigma like plant (2), RNA regulation of transcription (2), ABI3/VP1 family (2), C2C2 (Zn) Alfin-like (2), TCP (2), Methyl binding domain proteins (2), Nucleosome/chromatin (2), Polycomb Group (2), Zn-finger (CCHC) (2), GRP (1), GRF zinc finger (1), AT-rich interaction domain (1), AtSR (1), JUMONJI (1), NAC (1), NIN-like bZIP (1), PWWP domain protein (1), Silencing Group (1), SNF7 (1) and Pseudo ARR (1).

A total of 489 DEGs were involved in the second largest protein class group, including protein degradation ubiquitin (123), protein posttranslational modification kinase (73), synthesis. ribosomal protein (56), protein targeting unspecified (15), protein degradation (14), protein degradation cysteine protease (14), synthesis ribosomal biogenesis (14), protein synthesis initiation (14), protein degradation serine protease (11), protein degradation AAA type (8), protein synthesis elongation (7), protein degradation subtilizes (7), protein targeting nucleus (6), protein folding (6), protein targeting mitochondria (6), protein targeting chloroplast (4), protein targeting golgi pathway (4), protein targeting vacuole (4), protein targeting plasma membrane (2), protein targeting peroxisomes (2), protein targeting unknown (2), protein degradation autophagy (2), protein degradation metalloprotease (2), tyrosine-tRNA ligase (1), methionine-tRNA ligase (1), aspartate-tRNA ligase (1), cysteine-tRNA ligase (1), arginine-tRNA ligase (1), asparagine-tRNA ligase (1), bifunctional aminoacyl-tRNA synthetize (1), lysine-tRNA ligase (1), alanine-tRNA ligase (1), valine-tRNA ligase (1), activation (1), protein targeting ER pathway (1) and protein glycosylation (1).

**FIGURE 8:**
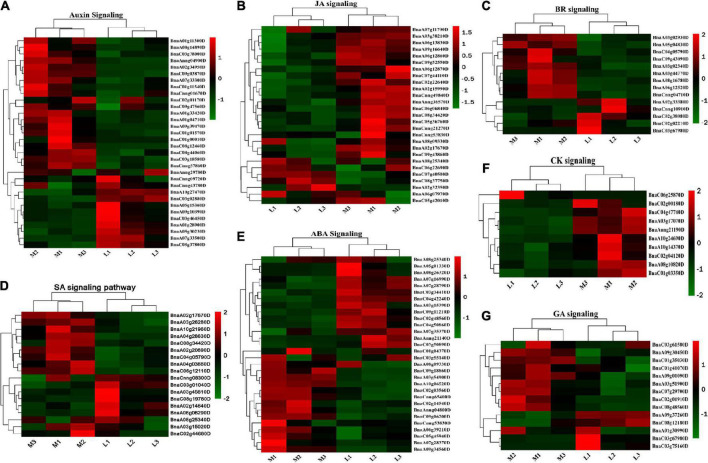
The demonstration of differentially expressed genes (DEGs) involved in the phytohormones pathway. **(A–G)** Heatmap of DEGs in ABA, JA, CK, SA, BR, IAA, and GA pathway. The sample and DEG no were shown on the bottom right of the figure, respectively. The color column in the legend represents the gene expression level.

It should be noted that a set of 74 DEGs were found to be associated with metabolic and signaling pathways of multiple hormones, including AUX (27), ETH (11), JA, (9), SA (9), BR (6), CK (5), ABA (4), and GA (3), showing the complex role and interactions of phytohormones (Figure 8). Of the 74 DEGs, many were associated with the synthesis or degradation of phytohormones, potentially contributing to the hormone concentration difference between more- and less-ovule lines (Supplementary Table 7).

To establish a link between the DEGs and ovule number, they were BLAST against the reported ovule number genes, mainly from *Arabidopsis* (Qadir et al., 2021). Of these DEGs 12 are homologous to known ovule number genes such as *CUC1* (*BnaA01g28990D*), *REV* (*BnaA02g06170D*), *CRF3* (*BnaA03g12320D*), *CRF6* (*BnaC06g42850D*), *CYP85A2* (*BnaC02g38080D*), *GA20OX* (*BnaC03g75160D*), *AP2* (*BnaCnng39690D*), *PAN* (*BnaC06g30310D*), *SHP1* (*BnaC08g29520D*), *CRC* (*BnaA07g27740D*), *INO* (*BnaC07g13110D*), and *HEMN1* (*BnaCnng15790D*). More than half of DEGs are involved in the know pathways of ovule number regulation, including development, phytohormones, transcription factors, protein, signaling (phosphorelay, sugar and nutrient physiology, G-protein and receptor kinase), and micro-RNA. These results highly suggested that the identified DEGs were highly associated with ovule number (Qadir et al., 2021).

### Identification of candidate genes through the integration of genome-wide association study and RNA-seq

To identify the candidate genes underlying the natural variation of ONPO in oilseed rape, a Venn diagram was constructed between 1,971 annotated genes within the genomic region of association loci and the 4,449 DEGs, which resulted in 73 overlapping genes (Supplementary Figure 5). Integrating functional annotation information, six genes underlying association loci on A03, A06, C01, and C06 chromosomes were selected, including *BnaA03g14600D, BnaA03g33420D, BnaA06g08920D, BnaA06g13210D, BnaC01g25840D*, and *BnaC03g16210D*.

## Discussion

In angiosperms, ovules are critical organs as they represent the direct progenitors of seeds (Endress, 2011; Qadir et al., 2021). Therefore, dissecting the genetic basis of ovule numbers will provide valuable information for the targeted improvement of seed crops’ yield (Banks et al., 2010; Qadir et al., 2021). In the present study, the GWAS, transcriptomic and physiological analyses were conducted to identify causal loci and candidate genes underlying ovule number variation in *B. napus*.

### Great potential for genetic improvement of ovule and seed number

The ONPO of the association population displayed a large variation from 19.2 to 43.8 (mean≈29) across the four investigated environments. This range (>20) was obviously larger than the previous reports (<13) in *B. napus* (Ali, 2018; Chen et al., 2019; Khan et al., 2019), which represented a valuable resource for the genetic improvement of this trait. It should be noted that the reported ONPO of oilseed cultivars (Li et al., 2014; Yang et al., 2017) was close to the mean of the association population, suggesting that this trait has hardly been selected during oilseed rape breeding. The large difference (>10) between the ONPO of current cultivars and the max of germplasm indicated the great potential for the genetic improvement of ONPO in oilseed rape.

It should be noted that the variance associated with genotype (8.073) was 21.3 times greater than that of the environment (0.379), suggesting that genotype rather than the environment is the primarily determinant of ovule number variation in oilseed rape. As expected, the calculated broad-sense heritability of ONPO in the current study (91.9%) was higher than those of most yield components and related traits reported in *B. napus* (Cai et al., 2014; Wang et al., 2020; Hussain et al., 2021), which suggested that it was mainly governed by genotype.

In addition, the seed-setting rates of 26 extreme lines were accurately calculated using ONPO and SNPS measured in the same experiment, which varied greatly from 29.8 to 75.1%, with a mean of 54.0%. Similarly, the seed-setting rate of 36 DH lines derived from C010 × C001 varied from 38.3 to 82.2%, with a mean of 56.1% (Chen et al., 2019). It should be noted that the reported seed-setting rates of oilseed rape cultivars, including ZY-50 (69.3%) and Zhongshuang11 (75.4%) were all larger than the corresponding mean of 26 extreme lines (Yang et al., 2017; Ali, 2018), which suggested that it should be strongly selected in breeding. The large gap between SNPS and ONPO highly indicated the great potential for the genetic improvement of seed number (Li et al., 2017).

### Novel loci associated with ovule number per ovary variation

Although a dozen linkage and/or association mapping studies have been conducted for seed number per silique in oilseed rape (Shi et al., 2015; Lu et al., 2017; Yang et al., 2017), rarely was about ovule number per ovary. Eight association loci were identified in an association panel of 521 oilseed rape accessions genotyped with the *Brassica* 60 K SNP array, which was distributed on A03, A09, A10, C02, C04, and C05 chromosomes, explaining 1.22–6.40% of phenotypic variance (Khan et al., 2019). Five QTLs were detected using a DH population of 180 lines derived from inbred lines 7–5 and ZY50, which were distributed on A03, A07, A10 and C06 chromosomes, explaining 1.9–17.38% of the phenotypic variance (Ali, 2018).

In the current study, a total of 18 association loci were identified using an association panel of 327 lines genotyped with *Brassica* 50 K SNP array. It should be noted that the physical positions of all these 18 association loci were different from the reported QTL of ovule number, suggesting all of them to be novel. More importantly, three association loci were repeatedly identified in the different environments, suggesting it to be the important target for maker-assisted selection (since breeder can’t see ovule number by eye) and further gene cloning.

### Physiological basis of ovule number variation

The previous studies showed that the ONPO was affected by flower size (Wetzstein et al., 2013; Cucinotta et al., 2020), flower position, nutrient availability (Chalcoff and Aizen, 2016), and phytohormones (Gomez et al., 2018; Barro-Trastoy et al., 2020). Therefore, several related physiological indexes (including flower order, leaf area and photosynthetic rate, and phytohormones content) were measured and compared between the M and L ovule lines.

Obviously, the ONPO of two representative extreme lines decreased with the bud positions from the bottom to top of the main inflorescence, which was consistent with a previous finding that ovule number decreased from the basal to distal of racemes in *Hosta ventricosa* (Cao et al., 2011). This result could be explained by the distance between the top flowers and the source organs of assimilates, which is highly accordant with the resource competition hypothesis. Whereas both leaf area and photosynthetic rates of more- and less-ovule lines have no significant difference between the more- and less-ovule lines, further study should focus on the leaf area index because it’s more representative of the resource. It has been well known that plant hormones play an important regulatory role in regulating ovule initiation and number (Barro-Trastoy et al., 2020; Cucinotta et al., 2020; Qadir et al., 2021; Yang and Tucker, 2021). In the present study, the contents of several types of endogenous phytohormones (ABA, BA, GA4, IAA, JA) have significant differences between more- and less-ovule lines. Highly accordant with this, the transcriptomic analysis identified 74 DEGs that are involved in the phytohormones pathway. More importantly, many DEGs are involved in the metabolic (such as synthesis and degradation) process of the same type of phytohormones (ABA, BR, CK, ETH, GA, IAA, JA, SA). In the future, it’s worth investigating the response of ovule number on different types of phytohormones using the representative lines in the oilseed rape crop.

### Association of identified deferentially expressed genes with ovule number

The 4,449 DEGs were enriched into a dozens of classes, including RNA (540), protein (489), signaling (196), transport (176), stress (121), cell (114), cell wall (97), photosynthesis (79), hormone metabolism (74), DNA (57), etc. This confirmed the complexity of the ovule initiation and development process at the transcriptome level. Of the 540 DEGs enriched in the largest class of RNA, most belonged to the transcription factors, which was consistent with the previous finding that Transcription factors (TFs) have been reported to play crucial roles in the reproductive development of flowering plants (Yang and Tucker, 2021). In addition, a total of 74 DEGs were involved in the metabolic and signaling pathway of several types of phytohormones, which was accordant with the different content of several types of phytohormones. Together, these results support the importance of phytohormones in ovule initiation and development (Barro-Trastoy et al., 2020; Qadir et al., 2021; Yang and Tucker, 2021; Yu et al., 2022). More importantly, about half of the DEGs belonged to the same functional category of the identified ovule number regulating genes. Especially, 12 DEGs are homologous to the known ovule number genes. These results highly suggested that the identified DEGs are associated with ovule number. Further studies are worthy of validating the function of the 12 homologous ovule number DEGs in oilseed rape by over-expression, RNAi, or gene editing.

### Candidate genes underlying ovule number per ovary variation

Integrating genome-wide association and transcriptome analysis is an efficient strategy for discovering candidate genes of complex traits (Li et al., 2020). In the current study, integrating association loci, DEGs and functional annotation, six candidate genes were identified, including *BnaA03g14600D, BnaA03g33420D, BnaA06g08920D, BnaA06g13210D, BnaC01g25840D*, and *BnaC03g16210D*. *BnaA03g33160D* is homologous to *Arabidopsis EIF4A1* that encodes RNA helicase, whose mutant decreased in both ovule number and fertility (Bush et al., 2015). *BnaA01g23050D* is homologous to *Arabidopsis HAP13*, which encodes μ1 adaptin component of the heterotetrameric protein complex that regulates protein sorting at the *trans*-Golgi network/early endosome. Its mutant displayed defects in outer integument growth as well as reduced ovule number (Wang et al., 2016). *BnaA03g33770D* is homologous to *Arabidopsis* transcription factor *CUC1*, which functions redundantly with *CUC2* and *CUC3* to regulate ovule initiation. The double mutant of *CUC1* and *CUC2* showed reduced ovule number (Cucinotta et al., 2018). These important candidate genes will be subjected to comparative sequencing and further functional validation in oilseed rape.

## Conclusion

In summary, this study investigated the genetic, physiological, and transcriptomic basis for the natural variation of ONPO in oilseed rape using an association population and extreme lines with more and less ovules. A wide variation in ovule number exists in oilseed rape, and this variation is primarily attributed to the genotype. Through the combination of genome-wide association and transcriptomic analysis, a total of 18 novel association loci and six candidate genes were identified, which provide a solid basis for maker-assisted selection and further gene cloning.

## Data availability statement

The datasets presented in this study can be found in online repositories. The names of the repository/repositories and accession number(s) can be found below: https://www.ncbi.nlm.nih.gov/, PRJNA820145.

## Author contributions

HW: formal analysis, funding acquisition, and supervision. JS: conceive and design the experiments, project administration, and revise the manuscript. MQ: perform the experiment, analyze the data, write the manuscript, and visualization. LQ: investigate the leaf area and photosynthetic rate. JY and NA: help to analyze the data. XW: resources and revise the manuscript. All authors contributed to the article and approved the submitted version.

## Footnotes

1
https://www.greenfafa.com/

2
http://www.Illumina.com/

3
https://www.ncbi.nlm.nih.gov/assembly/GCA_000751015.1

4
http://bar.utoronto.ca/ntools/cgi-bin/ntools_classification_superviewer.cgi
