# Supplementary material for: Genetic dissection of the natural variation of ovule number per ovary in oilseed rape germplasm (Brassica napus L.)
Source: Front Plant Sci. 2022 Sep 13;13:999790. doi: 10.3389/fpls.2022.999790 (PMC9513589; doi:10.3389/fpls.2022.999790)
Supplement: Supplementary file 1 [file Data_Sheet_1.docx]

Supplementary Material

**Supplementary Figure 1.** The QQ plot of GLM and MLM model in four investigated environments. The dotted lines indicated the significance threshold. The horizontal and vertical axes show expected and observed –log10 (P-value) for each SNP marker. The different combinations of model and environments were distinguished by the different colors as shown in the legends.

**Supplementary Figure 2.** Correlation between leaf area/photosynthetic rate and ovule number per ovary of 26 extreme lines. The horizontal and vertical axes show the ovule number per ovary and leaf area (A) and leaf photosynthetic rate (B) leaf photosynthetic rate, respectively. The positions of 26 dots shows the corresponding ovule number and leaf area and leaf photosynthetic rate. The 26 pairs of data were used to fit the trend line with R^2^.

**Supplementary Figure 3.** Statistics of gene expression in more- and less-ovule pools with three repeats. (A) The gene expression density in six samples. The horizontal and vertical axes show the log_10_(FPKM) and density respectively. (B) The Correlation between samples heat map (M1-3 vs. L1-3). The legend shows the distance from sample to sample, and the coefficients are displayed in color column. (C) Analysis of gene expression in each sample. The horizontal and vertical axes showed the sample and gene number respectively. The legends indicate the different pattern of gene expression FPKM (0-0.5; 0.5-1; 1-10 and > to 10).

**Supplementary Figure 4.** The qRT-PCR validation of 10 random DEGs. The horizontal and vertical axes represent the M/L pools and relative expression level respectively.

**Supplementary Figure 5.** The Venn diagram indicates the common genes by integration of DEGs and GWAS.
